# Supplementary material for: Effects of microbial-derived long-chain polyunsaturated fatty acids from Aurantiochytrium limacinum BCC52274 and Mortierella sp. on growth and immunity in Litopenaeus vannamei post-larvae
Source: PLoS One. 2025 Jul 31;20(7):e0329358. doi: 10.1371/journal.pone.0329358 (PMC12312968; doi:10.1371/journal.pone.0329358)
Supplement: S1 Table — (DOCX) [file pone.0329358.s001.docx]

**S1 Table.** Proportions of dried AL and ARACO used to supplement *Artemia* for the feed experiment.

| **Feed group** | **Ratio of total DHA:ARA in the emulsion** | **Weight of AL (mg/L)** | **Weight of ARASCO oil (mg/L)** |
| --- | --- | --- | --- |
| Control (R) | 0:0 | 0.00 | 0.00 |
| A | 100:0 | 403.05 | 0.00 |
| B | 75:25 | 302.05 | 63.38 |
| C | 50:50 | 201.52 | 126.76 |
| D | 25:75 | 100.76 | 190.14 |
| E | 0:100 | 0.00 | 253.52 |
