# Supplementary material for: Effects of microbial-derived long-chain polyunsaturated fatty acids from Aurantiochytrium limacinum BCC52274 and Mortierella sp. on growth and immunity in Litopenaeus vannamei post-larvae
Source: PLoS One. 2025 Jul 31;20(7):e0329358. doi: 10.1371/journal.pone.0329358 (PMC12312968; doi:10.1371/journal.pone.0329358)
Supplement: S2 Table — (DOCX) [file pone.0329358.s002.docx]

**S2 Table.** Primer sequences and PCR conditions for the qPCR analysis of shrimp immune genes in *L. vannamei*

| Genes | Abbreviation | Primer sequences | Tm (°C) | Product size (bp) | R^2^ | E |
| --- | --- | --- | --- | --- | --- | --- |
| Immune genes | | | | | | |
| *Prophenol oxidase I* | *ProPO-I* | F: 5’ ACGTCACTTCCGGCAAGCGA 3’ | 60 | 156 | 0.998 | 104.6 |
|  |  | R: 5’ CCTCCTTGT GAG CGTTGTCAGG 3’ |  |  |  |  |
| *Prophenol oxidase II* | *ProPO-II* | F: 5’ ACCACTGGCACTGGCACCTCGTCTA 3’ | 58 | 161 | 0.994 | 96.5 |
|  |  | R: 5’ TCGCCAGTTCTCGAGCTTCTGCAC 3’ |  |  |  |  |
| *Prophenoloxidase-activating enzyme* | *ppA* | F: 5’ CTAGAGACGTCGGTGTCATCACC 3’ | 62 | 151 | 0.999 | 100.5 |
|  |  | R: 5’ AACTTGCCGTCCGAAGTGCG 3’ |  |  |  |  |
| *Penaeidin 3a* | *PEN3a* | F: 5’ CGTGGTCTGCCTGGTCTTCTT 3’ | 61 | 112 | 0.997 | 95.0 |
|  |  | R: 5’ CAAAGGTCTCACGAAGGGTGGT 3’ |  |  |  |  |
| *Superoxide dismutase* | *SOD* | F: 5’ GCCCCAATCAGAACAAGC 3’ | 58 | 154 | 0.999 | 99.4 |
|  |  | R: 5’ AAGGCCTTCACGTAATCTGC 3’ |  |  |  |  |
