# Supplementary material for: Effects of microbial-derived long-chain polyunsaturated fatty acids from Aurantiochytrium limacinum BCC52274 and Mortierella sp. on growth and immunity in Litopenaeus vannamei post-larvae
Source: PLoS One. 2025 Jul 31;20(7):e0329358. doi: 10.1371/journal.pone.0329358 (PMC12312968; doi:10.1371/journal.pone.0329358)
Supplement: S3 Table — (DOCX) [file pone.0329358.s003.docx]

**S****3 Table** Fatty acid compositions and total fatty acid (TFA) contents (% dry weight) of *Artemia* supplemented with different ratios of DHA:ARA.

| **Fatty acids** | **R** | |  | **A** | |  | **B** | |  | **C** | |  | **D** | |  | **E** | |
| --- | --- | --- | --- | --- | --- | --- | --- | --- | --- | --- | --- | --- | --- | --- | --- | --- | --- |
|  | **%TFA** | **%DW** |  | **%TFA** | **%DW** |  | **%TFA** | **%DW** |  | **%TFA** | **%DW** |  | **%TFA** | **%DW** |  | **%TFA** | **%DW** |
| C14:0 | 3.70 | 0.08 |  | 3.47 | 0.31 |  | 3.55 | 0.11 |  | 3.57 | 0.08 |  | 3.64 | 0.08 |  | 3.72 | 0.08 |
| C15:0 | 4.31 | 0.05 |  | 6.20 | 0.12 |  | 4.47 | 0.06 |  | 4.19 | 0.05 |  | 4.22 | 0.05 |  | 4.32 | 0.05 |
| C16:0 | 1.30 | 1.36 |  | 1.29 | 4.70 |  | 1.37 | 1.85 |  | 1.47 | 1.38 |  | 1.49 | 1.37 |  | 1.52 | 1.39 |
| C18:0 | 3.31 | 0.73 |  | 4.80 | 0.72 |  | 3.27 | 0.72 |  | 2.86 | 0.69 |  | 2.83 | 0.71 |  | 2.84 | 0.71 |
| C22:0 | 42.74 | 0.03 |  | 29.11 | 0.04 |  | 39.96 | 0.03 |  | 42.70 | 0.04 |  | 42.51 | 0.04 |  | 40.87 | 0.04 |
| ∑SFA | 55.36 | 2.24 |  | 44.87 | 5.88 |  | 52.62 | 2.77 |  | 54.79 | 2.22 |  | 54.69 | 2.24 |  | 53.27 | 2.26 |
| C16:1 | 5.61 | 0.48 |  | 7.49 | 0.45 |  | 5.84 | 0.46 |  | 5.66 | 0.47 |  | 5.71 | 0.47 |  | 5.84 | 0.49 |
| C18:1 | 0.00 | 2.62 |  | 13.96 | 2.39 |  | 1.91 | 2.50 |  | 0.18 | 2.51 |  | 0.10 | 2.56 |  | 0.00 | 2.63 |
| C20:1 | 0.65 | 0.60 |  | 1.81 | 0.63 |  | 0.94 | 0.60 |  | 0.68 | 0.59 |  | 0.67 | 0.60 |  | 0.65 | 0.61 |
| ∑MUFA | 6.26 | 3.70 |  | 23.27 | 3.47 |  | 8.69 | 3.55 |  | 6.52 | 3.57 |  | 6.49 | 3.64 |  | 6.49 | 3.72 |
| C18:3 | 2R | 4.02 |  | 2A | 3.73 |  | 2B | 3.86 |  | 2C | 3.86 |  | 2D | 3.91 |  | 2E | 4.02 |
| C20:5 | 6.34 | 0.29 |  | 4.27 | 0.30 |  | 5.91 | 0.29 |  | 5.98 | 0.29 |  | 6.11 | 0.29 |  | 6.02 | 0.30 |
| C22:5 | 22.63 | nd |  | 14.16 | 0.43 |  | 20.56 | 0.06 |  | 21.93 | 0.01 |  | 22.12 | nd |  | 22.24 | nd |
| C22:6 | 7.71 | nd |  | 4.98 | 1.74 |  | 7.04 | 0.25 |  | 7.62 | 0.03 |  | 7.58 | 0.02 |  | 7.71 | nd |
| ∑n-3 | 36.68 | 4.31 |  | 23.41 | 6.20 |  | 33.50 | 4.47 |  | 35.53 | 4.19 |  | 35.82 | 4.22 |  | 35.96 | 4.32 |
| C18:2 | Control | 0.89 |  | 100:0 | 0.84 |  | 75:25 | 0.86 |  | 50:50 | 0.87 |  | 25:75 | 0.88 |  | 0:100 | 0.91 |
| C20:2 | 0.42 | 0.29 |  | 0.68 | 0.33 |  | 0.46 | 0.38 |  | 0.41 | 0.42 |  | 0.41 | 0.43 |  | 0.40 | 0.41 |
| C20:3 | 11.74 | nd |  | 27.91 | nd |  | 15.18 | nd |  | 12.03 | nd |  | 11.79 | nd |  | 11.75 | nd |
| C20:4 | 4.19 | 0.12 |  | 2.70 | 0.12 |  | 3.78 | 0.13 |  | 4.10 | 0.17 |  | 4.08 | 0.18 |  | 4.10 | 0.20 |
| ∑n-6 | 16.35 | 1.30 |  | 31.29 | 1.29 |  | 19.42 | 1.37 |  | 16.55 | 1.47 |  | 16.27 | 1.49 |  | 16.26 | 1.52 |
| ∑PUFA | 53.03 | 5.61 |  | 54.70 | 7.49 |  | 52.92 | 5.84 |  | 52.08 | 5.66 |  | 52.09 | 5.71 |  | 52.22 | 5.84 |
| ∑HUFA | 53.03 | 0.70 |  | 54.70 | 2.93 |  | 52.92 | 1.12 |  | 52.08 | 0.92 |  | 52.09 | 0.92 |  | 52.22 | 0.91 |
| n-3/n-6 | 2.24 | 3.31 |  | 0.75 | 4.80 |  | 1.73 | 3.27 |  | 2.15 | 2.86 |  | 2.20 | 2.83 |  | 2.21 | 2.84 |
| DHA/ARA | 1.84 | 0.00 |  | 1.84 | 13.96 |  | 1.86 | 1.91 |  | 1.86 | 0.18 |  | 1.86 | 0.10 |  | 1.88 | 0.00 |
| ∑TFA | 114.65 | 11.56 |  | 122.84 | 16.84 |  | 114.23 | 12.16 |  | 113.38 | 11.45 |  | 113.27 | 11.59 |  | 111.98 | 11.83 |
