# Supplementary material for: Effects of microbial-derived long-chain polyunsaturated fatty acids from Aurantiochytrium limacinum BCC52274 and Mortierella sp. on growth and immunity in Litopenaeus vannamei post-larvae
Source: PLoS One. 2025 Jul 31;20(7):e0329358. doi: 10.1371/journal.pone.0329358 (PMC12312968; doi:10.1371/journal.pone.0329358)
Supplement: Attachment — Submitted filename: A rebuttal letter.docx [file pone.0329358.s012.docx]

National Center for Genetic Engineering and Biotechnology

National Science and Technology Development Agency

Phahonyothin Road, Khlong Nueng, Khlong Luang

Pathum Thani, Thailand

13 July 2025

Dear Editor,

We are submitting a revised manuscript of our work titled “**Effects of microbial-derived long-chain polyunsaturated fatty acids from *Aurantiochytrium limacinum* BCC52274 and *Mortierella sp.* on growth and immunity in *Litopenaeus vannamei* post-larvae**” (PONE-D-25-31399) to be considered for research article publication in **PLOS ONE**. We have modified the manuscript based on the editor’s and reviewers’ comments. The point-by-point responses are provided below.

1. Please include the following items when submitting your revised manuscript:

- A rebuttal letter that responds to each point raised by the academic editor and reviewer(s). You should upload this letter as a separate file labeled 'Response to Reviewers'.
- A marked-up copy of your manuscript that highlights changes made to the original version. You should upload this as a separate file labeled 'Revised Manuscript with Track Changes'.
- An unmarked version of your revised paper without tracked changes. You should upload this as a separate file labeled 'Manuscript'.

**Answer:** We have provided a rebuttal letter (this document), a marked-up copy of our manuscript, and an unmarked version of the revised manuscript based on the journal’s instructions.

2. If you would like to make changes to your financial disclosure, please include your updated statement in your cover letter. Guidelines for resubmitting your figure files are available below the reviewer comments at the end of this letter.

**Answer:** We have made clarification regarding the financial disclosure in the second-to-last paragraph of the updated cover letter. Should you have any more questions, please let us know.

**Journal Requirements:**

When submitting your revision, we need you to address these additional requirements.

3. Please ensure that your manuscript meets PLOS ONE's style requirements, including those for file naming.

**Answer:** We have adjusted the manuscript so that it meets PLOS ONE’s style requirements in the revised version of the manuscript.

2. Thank you for stating the following financial disclosure:
“This research has received funding support from the Functional Ingredient & Food Innovation Program, National Center for Genetic Engineering and Biotechnology [grant number P19-51807] to VV and the NSRF via the Program Management Unit for Human Resources & Institutional Development, Research and Innovation [grant number B05F640184] to WW.”

Please state what role the funders took in the study. If the funders had no role, please state: "The funders had no role in study design, data collection and analysis, decision to publish, or preparation of the manuscript."
If this statement is not correct you must amend it as needed.
Please include this amended Role of Funder statement in your cover letter; we will change the online submission form on your behalf.

**Answer:** We have added the suggested sentence regarding the roles of the funders into the updated cover letter. Please change the online submission form on our behalf.

3. Please review your reference list to ensure that it is complete and correct. If you have cited papers that have been retracted, please include the rationale for doing so in the manuscript text, or remove these references and replace them with relevant current references. Any changes to the reference list should be mentioned in the rebuttal letter that accompanies your revised manuscript. If you need to cite a retracted article, indicate the article’s retracted status in the References list and also include a citation and full reference for the retraction notice.

**Answer:** We have rechecked all references by going through the journal’s database, google scholars, as well as databases for the retracted publications. However, we were unable to identify the retracted paper.

**Reviewer Comments to the Author**

**Reviewer #1**: Dear Author
Thank you for your good manuscript I accept it only with minor change
please add new references and revised again discussion .and short material and methods and explain more result and add main idea

**Answer:** We thank Reviewer #1 for your kind suggestions. As there was no specific instruction given in the reviewers’ response by PLOS ONE (which reference to add and which part of the discussion to be revised), we have improved our manuscript as follows:

1) Added new references can be found in line 416-419, 440-449, 479-481, and 496-498.
2) Revised the discussions: The discussion was revised to correct grammatical errors as well as the added discussion on the main idea (line 424-435) as well as more discussion based on the added new references shown in 1).
3) Short materials and methods: We have shortened the materials and methods as follows.

3.1) **Under the section pathogen challenge**, we have removed “to assess the effect of *Artemia* enrichment on the immunological phenotype of the PL in response to bacterial and viral infection, PL19 were challenged with *Vibrio harveyi* or white spot syndrome virus (WSSV). Details of the challenge are as follows.”

3.2) **Under the Quantitative real-time PCR analysis to detect WSSV copy number**, we have removed the following sentences.

“The water volume in each tank was adjusted from 150 L to 180 L on day 13 of the experiment to account for the larger size of the shrimp.”

“As WSSV infection in PLs typically does not result in mortality [33,34], the effect of feed supplementation on the WSSV-infected PLs was evaluated by determining the WSSV copy number using a qPCR analysis.”

“The extracted DNA was spectrophotometrically quantified (OD_260_) and qualified (OD_260_/OD_280_ ~ 1.6-1.9)”

and “Oligonucleotide primers were obtained from Integrated DNA Technologies in South Korea.”

4) Explain more results: We have added an explanation in the result section in line 280-282.
5) Added the main idea: A proposed model was added in line 410 to 435 as well as the added Conclusion section (line 507-518)

**Reviewer #2**: This study provides valuable insights into sustainable alternatives to fish oil in aquaculture feed by evaluating the efficacy of Aurantiochytrium limacinum BCC52274 (AL) and Mortierella sp. oil (ARASCO) as sources of long-chain polyunsaturated fatty acids (LC-PUFAs) for Pacific white shrimp (Litopenaeus vannamei) post-larvae. The experimental design, which involved Artemia enrichment with varying DHA:ARA ratios, effectively demonstrated that different LC-PUFA profiles can be strategically applied to promote either growth or immune enhancement in shrimp. Notably, Group A (DHA:ARA 100:0) achieved the highest biomass and body weight, while Group D (DHA:ARA 25:75) exhibited elevated prophenoloxidase II expression and increased production of anti-inflammatory eicosanoids. Furthermore, several enriched groups showed a reduced viral load following WSSV infection, highlighting the immunoprotective potential of these dietary supplements. Overall, the findings support the viability of AL and ARASCO as sustainable, functional replacements for traditional marine-derived fatty acids in shrimp aquaculture. The manuscript is well-written and well-structured, with only a few minor comments for consideration.

**Answer**: We thank the reviewer for your kind comments. We have clarified the manuscript based on the comments provided in the following section.

**Minor Comments:**

1. Lines 211–217: Please specify the sample size and the age of the post-larvae used for RNA extraction.

**Answer:** PL18 (*N* = 30) were used for RNA extraction to determine the impact of DHA:ARA supplementation on shrimp immune genes. We have added this information to line 217. For the consistency of the manuscript, we have also added the information regarding the PL sample size in each rearing tank (*N* = 15,000) in line 139-140 and the number of PL used to determine WSSV copy number (*N* = 35) in line 173.

2. The concentration of RNA used for cDNA synthesis should be mentioned.

**Answer**: The author has added the concentration of RNA used for the cDNA synthesis in lines 222 along with the amounts of cDNA used in each real-time PCR reaction in lines 229.

3. *Vibrio harveyi* is not mentioned in the abstract and should be included.

**Answer**: The authors have included a sentence regarding the *V. harveyi* experiment in the abstract (lines 48-49).

4. The discussion section would benefit from a deeper analysis of the findings in the context of existing literature, or by proposing hypotheses where data are currently lacking.

**Answer**: We have added a proposed model and more discussion in line 419-431, 490-496, and 500-511.

One of the references in the discussion section “The supplementation of 0.5 and 1.5% of Trichoderma harzianum, a microfungus isolated from rivers and streams in Indonesia, improved survival and growth performance in *L. vannamei* PLs after 18 days of feeding [44]” was removed as there are better references that are better related to the subject of this manuscript.

We thank both the academic editor and reviewers for their invaluable comments that help improve the quality of our manuscript. If you would like further clarification or modification of the manuscript, please let us know. We are looking forward to hearing a positive response from you.

Wananit Wimuttisuk

[wananit.wim@biotec.or.th](mailto:wananit.wim@biotec.or.th)

National Science and Technology Development Agency, Thailand
